# Supplementary material for: Resolving the full spectrum of human genome variation using Linked-Reads
Source: Genome Res. 2019 Apr;29(4):635–45. doi: 10.1101/gr.234443.118 (PMC6442396; doi:10.1101/gr.234443.118)
Supplement: Supplemental Material [file supp_gr.234443.118_Supplemental_longranger-2.2.zip › longranger-2.2.tar.bz2!/lr2.2-public/lib/stan/cmdstan/stan/doc/api/html/structstan_1_1agrad_1_1fvar-members.html]

Stan: Member List


|  |  |
| --- | --- |
|  | Stan  2.5.0  probability, sampling & optimization |


- Main Page
- Related Pages
- Namespaces
- Classes
- Files

- Class List
- Class Index
- Class Hierarchy
- Class Members

All Classes Namespaces Files Functions Variables Typedefs Enumerations Enumerator Friends Macros Pages

- stan
- agrad
- fvar

stan::agrad::fvar< T > Member List

This is the complete list of members for stan::agrad::fvar< T >, including all inherited members.

|  |  |  |
| --- | --- | --- |
| d\_ | stan::agrad::fvar< T > |  |
| fvar(const TV &val, const TD &deriv) | stan::agrad::fvar< T > | inline |
| fvar(const TV &val) | stan::agrad::fvar< T > | inline |
| fvar() | stan::agrad::fvar< T > | inline |
| operator\*=(const fvar< T2 > &x2) | stan::agrad::fvar< T > | inline |
| operator\*=(const T2 &x2) | stan::agrad::fvar< T > | inline |
| operator++() | stan::agrad::fvar< T > | inline |
| operator++(int) | stan::agrad::fvar< T > | inline |
| operator+=(const fvar< T2 > &x2) | stan::agrad::fvar< T > | inline |
| operator+=(const T2 &x2) | stan::agrad::fvar< T > | inline |
| operator--() | stan::agrad::fvar< T > | inline |
| operator--(int) | stan::agrad::fvar< T > | inline |
| operator-=(const fvar< T2 > &x2) | stan::agrad::fvar< T > | inline |
| operator-=(const T2 &x2) | stan::agrad::fvar< T > | inline |
| operator/=(const fvar< T2 > &x2) | stan::agrad::fvar< T > | inline |
| operator/=(const T2 &x2) | stan::agrad::fvar< T > | inline |
| operator<<(std::ostream &os, const fvar< T > &v) | stan::agrad::fvar< T > | friend |
| tangent() | stan::agrad::fvar< T > | inline |
| val() | stan::agrad::fvar< T > | inline |
| val\_ | stan::agrad::fvar< T > |  |
| value\_type typedef | stan::agrad::fvar< T > |  |


---

[ Stan Home Page ]

*© 2011–2014,
Stan Development Team.*
